# Thermal Ecology and Homeostasis in Colonies of the Neotropical Arboricolous Ant *Azteca chartifex spiriti* (Formicidae: Dolichoderinae)

**DOI:** 10.3390/insects17010032

**Published:** 2025-12-25

**Authors:** Josieia Teixeira dos Santos, Elmo Borges de Azevedo Koch, Julya Lopes dos Santos, Laís da Silva Bomfim, Jacques Hubert Charles Delabie, Cléa dos Santos Ferreira Mariano

**Affiliations:** 1 Programa de Pós-Graduação em Zoologia, Universidade Estadual de Santa Cruz (UESC), Km 16, Rodovia Jorge Amado (BR-415), Ilhéus 45662-900, BA, Brazil; josieiabiologa@gmail.com (J.T.d.S.); lsbomfim.ppgzoo@uesc.br (L.d.S.B.); csfmariano@uesc.br (C.d.S.F.M.); 2 Departamento de Ciências Biológicas, Universidade Estadual de Feira de Santana (UEFS), Avenida Transnordestina s/n, Feira de Santana 44036-900, BA, Brazil; elmoborges@gmail.com; 3 Curso de Licenciatura em Biologia, Universidade Estadual de Santa Cruz (UESC), Km 16, Rodovia Jorge Amado (BR-415), Ilhéus 45662-900, BA, Brazil; jlsantos.lbi@uesc.br; 4 Laboratório de Mirmecologia, Comissão Executiva do Plano da Lavoura Cacaueira (CEPLAC), Centro de Pesquisas do Cacau (CEPEC), Km 22, Rodovia Jorge Amado (BR-415), Ilhéus 45662-902, BA, Brazil

**Keywords:** homeostasis, foraging, moisture, nest architecture, polymorphism

## Abstract

Arboreal ants occupy a thermally dynamic environment, yet the mechanisms integrating nest architecture and worker behavior to maintain colony homeostasis remain understudied. We investigated the interplay among circadian rhythm, nest homeostasis, and worker morphology in *Azteca chartifex spiriti*, a Neotropical arboreal species that builds large polydomous nests suspended in trees. In ten colonies, we measured internal moisture and temperature gradients in the main nest, which houses most individuals, including the reproductive female, immatures, and numerous workers. In six colonies, we assessed the polymorphism of foraging workers over a 24 h cycle in relation to external temperature variation. The results show integrated thermoregulatory mechanisms that combine passive strategies, derived from nest architecture and moisture gradients from the suspension base to the lower extremity, with active strategies linked to foraging patterns and worker polymorphism. Internal temperature (27.8 ± 2.41 °C) remained buffered relative to external fluctuations, and moisture was significantly higher at the nest’s lower extremity (*p* < 0.001). Worker size displayed a bimodal distribution during the day that shifted to a unimodal pattern at night, indicating behavioral adjustments to thermal and operational demands. These findings demonstrate that the interaction between physical structure and worker behavior maintains colony homeostasis, providing essential insights into how dominant canopy ants may cope with future climate change scenarios.

## Simple Summary

Ant colonies require a stable internal environment to thrive, yet they must also adapt to daily fluctuations in temperature and humidity. We studied a tropical ant species that constructs large paper-like nests in trees to explore how daily activity, size variations among workers, and nest structure contribute to the colony’s well-being. We measured temperature and moisture levels within several nests and observed worker behavior throughout the day. We found that the nest structure effectively buffered internal conditions against external weather changes, with moisture levels varying from the top to the bottom of the nest. These factors influenced the ants’ distribution and the variation in heat within the structure. Additionally, we found that ants of different sizes were active at distinct times of day, suggesting that workers adjust their roles to meet the colony’s changing needs. This interplay of nest design, internal conditions, and adaptable behavior enables these ants to maintain a safe environment for both brood and adults, helping the colony cope with fluctuating climatic conditions. This understanding enhances our knowledge of how social insects manage environmental challenges and may inspire innovative approaches to sustainable temperature control in human structures.

## 1. Introduction

*Azteca chartifex spiriti* Forel, 1912, dominates the tree canopy where it nests, contributing to the mosaic of dominant arboreal ant species in the cocoa-producing region of Bahia, Brazil [1]. Like other species of the genus, it exhibits a polydomous nesting system (where a single colony occupies multiple nests) [2], and its colonies can contain thousands of highly aggressive workers, which play a crucial ecological role in shaping the composition of canopy arthropod communities [1,3,4]. Evidence indicates that this species has strong potential as a biological control agent due to three key traits: (i) its presence is linked to reduced populations of harmful insects in cocoa plantations [5,6]; (ii) its colonies demonstrate spatial and temporal stability [7]; and (iii) they exhibit a high capacity for territorial expansion [1,6,7]. Due to these characteristics, *A. chartifex spiriti* emerges as a promising model for studies on integrated pest management and the conservation of ecosystem services provided by cocoa agroforestry systems. However, understanding its thermal ecology is essential, as this species faces specific thermoregulatory challenges inherent to its arboreal habitat and constant exposure to fluctuating wind, rainfall, and solar radiation.

Ants represent one of the most abundant and ecologically significant groups of terrestrial invertebrates, exhibiting wide geographic distribution and occurrence across diverse environments, which reflects their high taxonomic and functional diversity [8,9]. Their remarkable behavioral and ecological diversity includes complex collective behaviors that ensure colony homeostasis, a key factor for survival under varying environmental conditions. Among these behaviors, thermoregulation stands out as a fundamental mechanism that integrates nest architecture and social behavior to maintain stable microclimatic conditions within colonies [10,11,12]. Various species display distinct thermoregulatory strategies, including *Solenopsis invicta* [13], *Acromyrmex heyeri* [14], and *Eciton burchellii* [15], as well as other hypogeic ants [12,16,17,18]. In addition to temperature, humidity gradients and behavioral plasticity also shape nest architecture and worker spatial distribution [12].

The reproductive cycle and the development of immature stages strongly depend on the thermal homeostasis within the nest, prompting the colony to adopt various strategies to maintain this homeostasis [19,20,21]. Adaptive responses such as thermal plasticity [22,23,24,25] and temporal niche partitioning [25,26,27] allow colonies to adjust their foraging patterns [28,29,30] to optimal thermal conditions [31,32,33]. Moreover, mature colonies exhibit morphological variation among workers, which promotes task specialization, including foraging and maintenance of the internal microclimate [34,35,36,37]. These behaviors are often regulated by circadian rhythms, which drive ants to adjust their activities in response to nycthemeral (day-night) cycles [38,39,40,41].

Arboreal colonies may be particularly vulnerable to extreme thermal events and shifts in temperature regimes [42], as physiological limits constrain foraging activity and brood development. Microclimatic studies indicate that solar radiation and structural complexity generate spatial and temporal thermal variations, influencing foraging windows [43,44,45]. Despite the recognized ecological importance of ants, the specific influence of abiotic factors on the activity of arboreal species remains less studied compared to ground-dwelling species [43,46]. This gap highlights the urgent need for studies integrating nest architecture, microclimatology, and behavioral ecology to guide conservation strategies [47].

We hypothesized that *A. chartifex spiriti* colonies utilize a combination of nest architectural features (passive control) and shifts in worker foraging activity (active control) to buffer the colony against external thermal fluctuations. Based on this, this study aimed to: (i) examine how environmental thermal fluctuations influence nest ecology; (ii) identify the thermal control mechanisms employed by colonies to maintain internal thermal homeostasis; (iii) assess the role of worker polymorphism in colony thermoregulation; and (iv) analyze how the physical and structural properties of nests contribute to the thermal regulation of colonies.

## 2. Materials and Methods

### 2.1. Study Area

**Figure 1:**
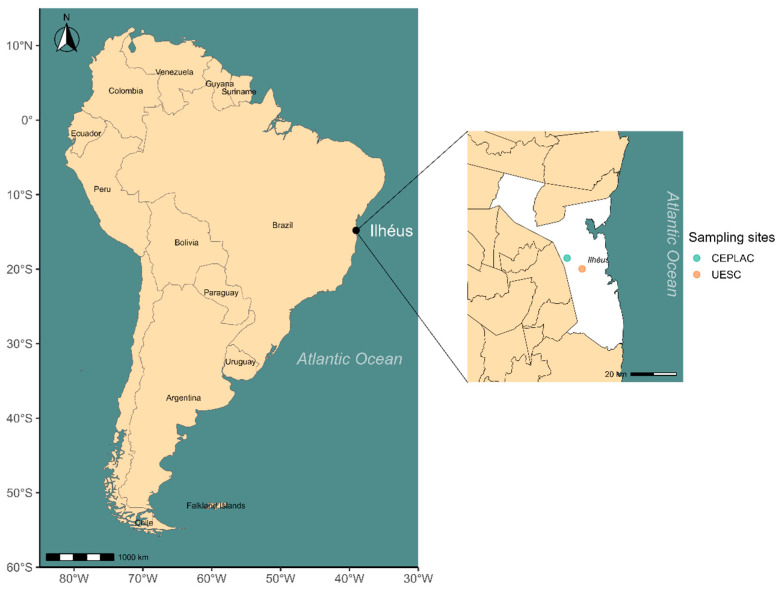
Map of South America highlighting the city of Ilhéus, located on the southern coast of the state of Bahia, Brazil. The map was created using public geospatial data to represent the study area where fauna associated with ant nests were sampled and analyzed.

This study was conducted in the municipality of Ilhéus, Bahia, Brazil (Figure 1). Located within the Atlantic Forest biome, the region exhibits a diverse vegetation structure, including “restinga” formations, mangrove forests, cacao plantations, wetlands, and floodplains [48]. The climate is humid tropical, with mean annual temperatures ranging from 20 °C to 25 °C and precipitation exceeding 1200 mm.

Colonies of *Azteca chartifex spiriti* were collected in agroforestry cacao systems (for definitions of the different types of cacao cultivation in the region, see [49]) (i) on the campus of the Universidade Estadual de Santa Cruz (UESC) (14°47′44″ S, 39°10′06″ W), characterized by a “cabruca” system, where cacao trees are cultivated under the shade of the remnant Atlantic Forest canopy; and (ii) in the experimental areas of the Comissão Executiva do Plano da Lavoura Cacaueira (CEPLAC) (14°46′58″ S, 39°13′16″ W), representing a clear-cut cultivation system lacking arboreal cover.

### 2.2. Experimental Design

To investigate the interrelationship among the nycthemeral rhythm, nest homeostasis, and worker morphometry within the polydomous nesting system of *Azteca chartifex spiriti*, the study was structured into two complementary experimental phases. The first focused on the internal humidity gradient of the nests, while the second evaluated how external temperature fluctuations influence nest temperature and worker polymorphism under various activity conditions.

#### 2.2.1. Phase I: Internal Nest Humidity Patterns

**Figure 2:**
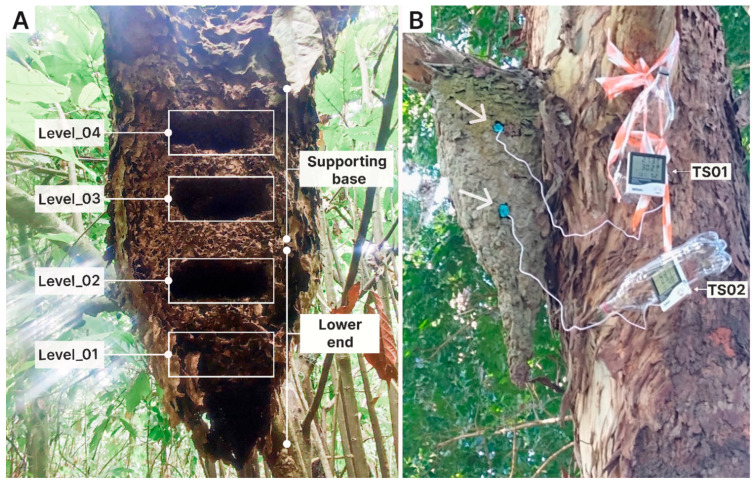
Examples of *Azteca chartifex spiriti* nests located on the campus of the State University of Santa Cruz (UESC). (**A**) Vertical division of nests into sampling levels; white rectangles indicate the points at which nest material was collected at each level. (**B**) Installation of thermal sensors (TS) within the nests; white arrows indicate the positions of the two sensors (TS01 and TS02) installed on the nest structure.

Ten polydomous colonies were selected, each with a main nest that exceeds 60 cm in height. Only the principal nest from each colony was used in this phase. In each nest, four vertical layers were defined, each 15 cm thick, establishing a vertical sampling gradient (from bottom to top) across four levels: from level 1, at the lower extremity, to level 4, at the base where the nest is attached to the supporting branch (Figure 2A).

From each level, samples of the nest’s construction material were collected, fragmented, and stored in glass beakers of 100 mL. Samples were first weighed fresh using an analytical balance (APX200/Denver Instrument Company, Arvada, CO, USA) and subsequently oven-dried (Tecnal TE-393/2, Piracicaba, Brazil) at 45 °C for 72 h before they were reweighed. The moisture percentage for each level was calculated based on the difference between wet and dry weights.

#### 2.2.2. Phase II: Thermal Ecology and Worker Morphometry

Six polydomous colonies were selected, each with a main nest taller than 60 cm in height. Two digital temperature sensors (Exbom FEPRO-MUT60OS, Exbom Hong Kong Co., Hong Kong, China, accuracy ± 1 °C) were installed in each main nest to simultaneously monitor internal temperature: one at the basal attachment point and another at the lower extremity (Figure 2B). After a 40 min acclimation period to allow for sensor calibration and worker adjustment, temperature monitoring was conducted continuously for 24 h, from 15:00 on one day to 15:00 the following day.

Internal temperatures (from both the base and lower extremity) and external ambient temperatures were recorded every two hours, resulting in a total of 13 readings per colony across the six monitored colonies. Sampling times were categorized as morning (05:00–11:00), afternoon (13:00–17:00), and night (19:00–03:00 the following morning).

Simultaneously with thermal monitoring, five foraging workers were randomly collected from the trunk of the supporting tree every two hours. Workers were preserved and measured under a trinocular stereomicroscope (LEICA DMC2900, Wetzlar, Germany) equipped with Leica Application Suite (LAS V4.5 software, released in November 2014). The following morphometric traits (in millimeters) were analyzed: (i) head width, which serves as a proxy for body size and strength; (ii) Weber’s length, which is the largest rigid body measurement, which serves as an indicator of body size and metabolic traits [50]; and (iii) femur length of one hind leg, which serves as a proxy for foraging speed and thermoregulatory strategy [51]. In total, 390 workers were used in this part of the experiment: six colonies × 13 samples of five workers collected every two hours.

### 2.3. Statistical Analysis

Moisture distribution in the main nest was assessed using the Kruskal–Wallis test. Sampling levels were defined as the predictor variable, and moisture content as the response variable. Pairwise comparisons were conducted using Dunn’s test (1964). Differences in the internal temperature of the main nest were evaluated using Student’s t-test for independent samples, comparing: (i) the temperature at the supporting base versus the lower end of the structure; and (ii) the internal nest temperature versus the external ambient temperature. Diurnal variation in temperature was investigated using analysis of variance (ANOVA).

Worker polymorphism across different activities and colonies was examined by correlating morphological parameters (head width, femur length and Weber’s length) using Pearson’s rank correlation coefficient. The relationship between foraging activity and worker polymorphism was tested with the Kruskal–Wallis test, followed by multiple comparisons using Dunn’s test (1964). In this analysis, the morphological parameters were treated as predictor variables and the periods of the day as the response variable. All statistical analyses and figures were produced with R v. 4.5.0 (R Core Team, 2025).

## 3. Results

### 3.1. Phase I: Internal Nest Humidity Patterns

**Figure 3:**
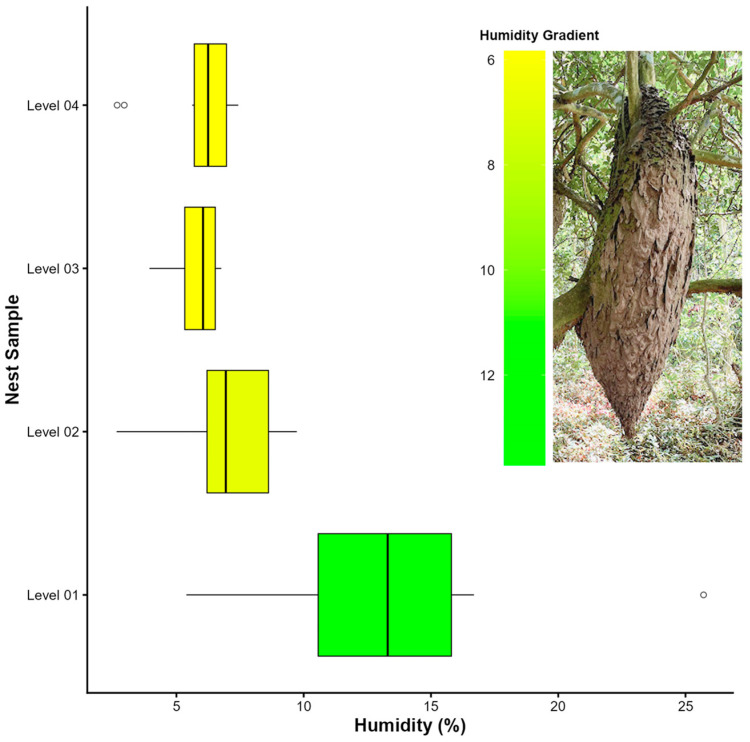
Boxplots showing variation in moisture content across the four sampling levels in nests of *Azteca chartifex spiriti*. Boxes represent the median and interquartile range; whiskers indicate the range of the data while excluding outliers, and individual points denote discrepant observations.

The studied colonies exhibited a consistent pattern in moisture distribution: the lower end of the main nest showed consistently higher moisture content than the upper portion of the nest that attaches to the supporting branch. A significant difference in moisture content was found among sampling levels (Kruskal–Wallis: H = 17.61; df = 3; *p* < 0.001). Pairwise comparisons using Dunn’s test indicated that level 1 differed significantly from level 3 (Z = 3.90; *p* < 0.001) and level 4 (Z = 3.28; *p* < 0.05). Level 2 did not show significant differences when compared to the other levels (Figure 3).

**Figure 4:**
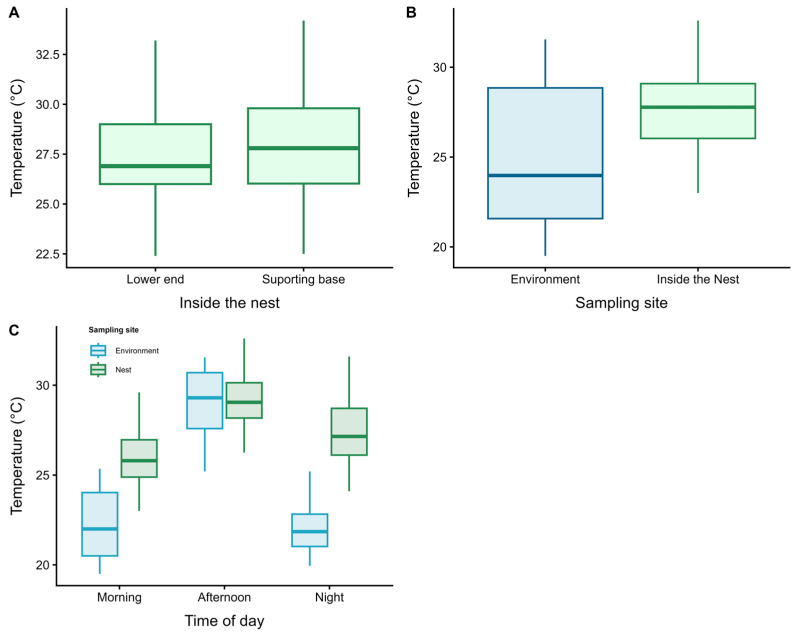
Comparison of mean temperatures: (**A**) between the supporting base and the lower end of the nest; (**B**) between internal nest temperature and external ambient temperature; (**C**) among different periods of the day. Bars indicate the means and the corresponding error bars. Statistical tests: Student’s *t*-test for (**A**,**B**) and ANOVA for (**C**).

**Figure 5:**
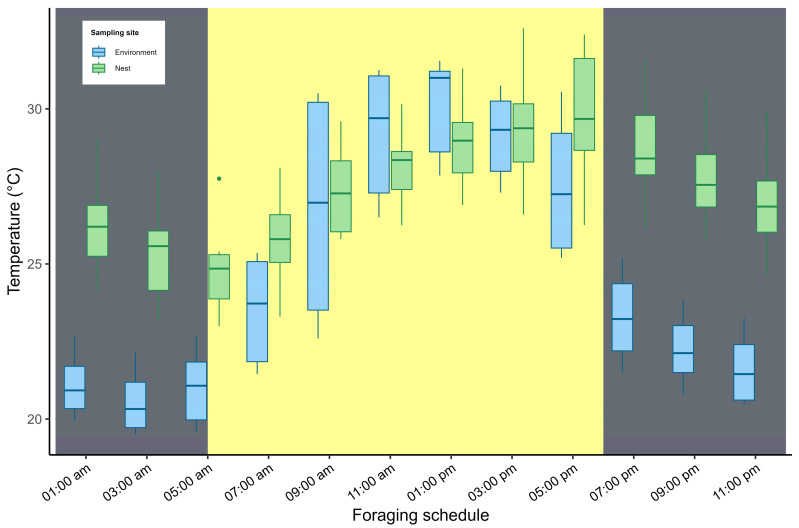
Diurnal variation in temperature: comparison between the internal nest temperature and external ambient temperature. Vertical bars indicate the boundaries between the daytime period (shown in yellow) and the nighttime period (shown in gray). Twilight periods are quite short (approximately 20 min) in the study area.

Internal nest temperature showed homogeneous variation between the supporting base (mean = 27.8 ± 2.41 °C) and the lower end (mean = 27.3 ± 2.40 °C), with no significant difference between these points (t = −1.48; df = 154; *p* = 0.14; Figure 4A). In contrast, a significant difference was found between internal nest temperature and external ambient temperature (t = −5.06; df = 123; *p* < 0.001; Figure 4B). Significant thermal variation was also observed across different periods of the day (ANOVA: F_2,153_ = 51.06; R^2^ = 0.40; *p* < 0.001; Figure 4C). Internal nest temperature tended to increase in parallel with rising external ambient temperature (linear model: F_11,144_ = 83.6; R^2^ = 0.46; *p* < 0.001; Figure 5).

### 3.2. Phase II: Thermal Ecology and Worker Morphometry

**Figure 6:**
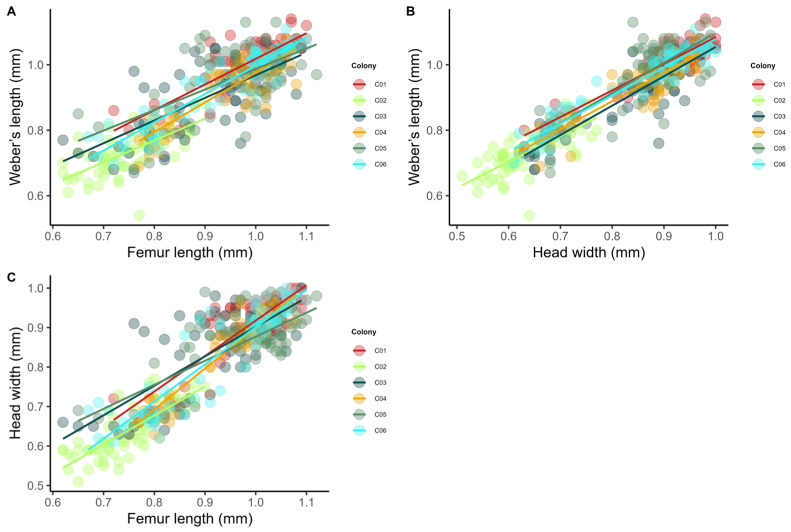
Correlations among morphological parameters of *Azteca chartifex spiriti* workers: (**A**) Weber’s length vs. femur length; (**B**) Weber’s length vs. head width; (**C**) head width vs. femur length. Points represent individuals, and colors characterize the colonies analyzed (C01–C06).

In the preliminary study of worker polymorphism, strong correlations were observed between Weber’s length and hind femur length (r = 0.86, *p* < 0.001) (Figure 6A), and between Weber’s length and head width (r = 0.92, *p* < 0.001) (Figure 6B). In addition, head width was strongly correlated with hind femur length (r = 0.89, *p* < 0.001) (Figure 6C).

**Figure 7:**
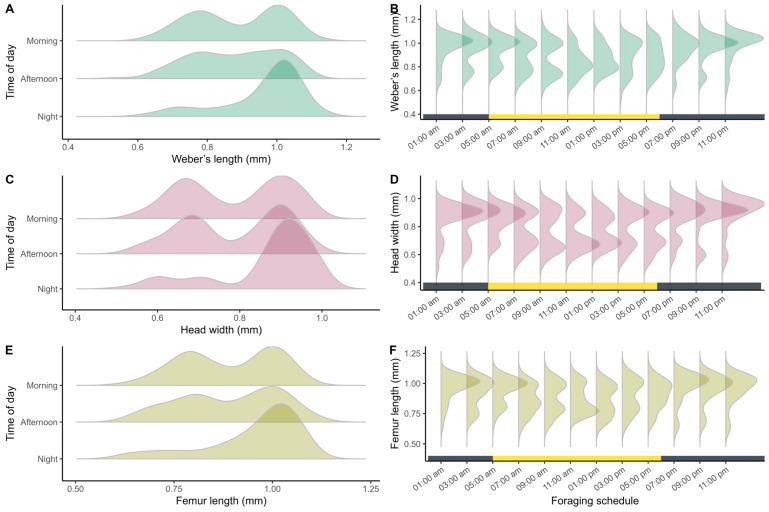
Distribution of variation in morphological traits of *Azteca chartifex spiriti* workers during foraging periods and across the daily cycle. (**A**,**B**) Weber’s length; (**C**,**D**) head width; (**E**,**F**) hind femur length. Each point represents an individual sampled in different activity periods; the lower horizontal bar indicates the alternation between the daytime period (yellow) and the nighttime period (gray).

Significant differences in worker size were found among different periods of the day for all three traits analyzed: Weber’s length (Kruskal–Wallis: H = 36.6; df = 2; *p* < 0.001; Figure 7A), head width (H = 41.84; df = 2; *p* < 0.001; Figure 7C), and hind femur length (H = 27.6; df = 2; *p* < 0.001; Figure 7E). Multiple comparisons (Table S2, Supplementary Materials) indicate that workers active during the night differ significantly from those active during other periods for all variables.

Based on the observed morphometric patterns, two distinct foraging patterns across the daily cycle were identified. During the night period, between 07:00 PM and 03:00 AM, foraging activity followed a unimodal pattern characterized by a predominance of larger workers (Figure 7B,D,F). During the daytime period, between 05:00 AM and 05:00 PM, foraging exhibited a bimodal structure that remained consistent throughout the day, indicating a greater diversity in the sizes of workers involved in these activities (Figure 7B,D,F).

## 4. Discussion

### 4.1. Phase I: Internal Nest Humidity Patterns

Our results indicate that temperature is evenly distributed within the main nest, with no significant differences between the basal attachment and the lower extremity, indicating a thermally stable microclimate. However, the moisture content was significantly higher in the lower portion of the nest. This variation is clearly influenced by nest morphology: while the basal attachment is broader and firmly anchored to the tree trunk, the lower extremity is narrower and elongated, creating a gradient that promotes the drainage of water collected by the structure (from dew, ambient humidity, or rainfall). Moreover, being positioned beneath a wide supporting branch, this portion remains partially shielded from direct solar radiation and wind exposure. Additionally, the network of interconnected tunnels and internal chambers that are linked by multiple openings establishes a natural ventilation system that facilitates the inflow of fresh air and the expulsion of air saturated by moisture. This architectural configuration contributes to maintaining a stable internal microclimate suitable for colony homeostasis, as also observed in other social insects whose nest design plays a crucial role in thermal regulation and gas exchange [14,52,53]. These findings reinforce the idea that nest architecture, whether subterranean or arboreal, constitutes a fundamental component of the colony’s ability to buffer environmental fluctuations and maintain physiological homeostasis [52].

The insulating properties of the nest-building material represent another crucial factor for maintaining internal microclimate stability. Many *Azteca* species construct carton nests using a combination of sediments and cellulose fibers cemented by proteinaceous secretions from the workers’ maxillary glands [54]. These structural components are also found in the nests of other arboreal ants, such as *Oecophylla*, *Camponotus* and *Polyrhachis* [44,55,56], as well as in arboreal termites [57] and social wasp colonies [58]. Cellulose fibers provide mechanical strength to the nest, whereas the proteinaceous compounds secreted by workers form a hydrophobic barrier that reduces permeability and shields the nest’s interior from excessive moisture. These insulating properties are essential for maintaining the microclimatic stability of the colonies, ensuring favorable conditions for survival, brood development, and overall colony functioning [59].

The diurnal variation in temperature shows that during the afternoon period, the internal temperature of the nests tends to rise with increases in ambient temperature. Passive strategies may explain the colony’s ability to maintain a stable microclimate even during periods of higher heat. In addition to nest architecture, the thermal diffusivity of the building material, combined with the humidity gradient, likely functions as an important thermal moderator, preventing colony overheating.

Similarly to the findings of Klingner [60] in the paper wasp nests, fluctuations in internal humidity may create pressure gradients between the intranidal and external environments, which are dissipated through the nest envelope and contribute to thermal stabilization. Another relevant mechanism is evaporation, an endothermic process that aids in temperature reduction. In *A. chartifex spiriti* nests, a comparable process may occur, where external air cools upon contact with the internal humidity of the nest, establishing a convective flow that helps maintain a cooler microclimate during temperature peaks.

In his thermodynamic analysis of *Formica polyctena* nests, Frouz [61] emphasized the central role of humidity in thermal regulation, noting contrasting effects between dry and moist nests. Additionally, Frouz [61] and Kadochová [11] demonstrated that nest humidity can influence microbial activity, increasing internal temperature or altering the insulating properties of the material, resulting in greater heat dissipation. Therefore, humidity not only modulates temperature but also determines the overall thermal balance within the nest, depending on both internal and environmental conditions.

### 4.2. Phase II: Thermal Ecology and Worker Morphometry

Despite the temperature reduction observed during the morning and nighttime periods, both inside and outside the nests, the intranidal temperature remained consistently higher than that of the external environment. Considering foraging patterns, worker polymorphism, and thermal fluctuations, it can be inferred that metabolic heat is the primary mechanism responsible for maintaining the temperature of the nest during periods of low external temperature. This heat, generated by the metabolic and behavioral activities of the organisms within the nest, acts as a key thermal modulator, ensuring the stability of the internal climate [15,62,63].

Studies conducted by Frouz [59] on *Formica polyctena* nests and by Korb [64] on *Macrotermes bellicosus* (Macrotermitinae) demonstrated the relevance of associated microorganisms in nest thermoregulation. In this context, *A. chartifex spiriti* stands out among arboreal ants because, in addition to hosting a highly diverse associated fauna [65,66,67], its colonies are exceptionally large and populous, containing tens of thousands of workers [67,68,69]. This high population density, combined with the continuous activity of workers, contributes to the generation and retention of metabolic heat, ensuring thermal homeostasis even under adverse environmental conditions.

The worker caste polymorphism in *A. chartifex spiriti*, previously documented by Wheeler [67] and Longino [70], may serve as an active thermoregulatory mechanism for the colony. Miranda [69] provided a detailed characterization of the spatial organization and population structure of this species, demonstrating that the polymorphic worker caste is functionally specialized within the nest. Larger workers perform defensive and maintenance activities, while smaller workers attend to the brood and maintain internal conditions. This segregation of tasks suggests that worker polymorphism is not only a behavioral adaptation linked to territorial dominance and resource defense, but may also indirectly contribute to microclimatic regulation. In the present study, polymorphic patterns were analyzed to support the hypothesis that metabolic heat and the spatial division of labor jointly mediate nest thermal stability during the nighttime period. This segregation of tasks suggests that worker polymorphism is not only a behavioral adaptation linked to territorial dominance but is also intimately linked to the colony’s interaction with the environment. In the present study, we observed a distinct shift in foraging patterns: a bimodal size distribution during the day and a unimodal pattern (larger workers) at night. While we initially hypothesized that the activity of larger workers during the night might actively contribute to thermal maintenance via metabolic heat, we must also consider that environmental conditions may act as a filter for worker activity. Larger workers, having a lower surface-area-to-volume ratio, may have different thermal inertia or desiccation resistance compared to smaller workers, allowing them to forage more effectively under specific nocturnal conditions [71,72]. Alternatively, ecological factors unrelated to temperature could drive this pattern. For instance, larger workers might be more efficient at defending against nocturnal predators or transporting specific resources available at night, as predicted by the size-grain hypothesis [51]. Therefore, the observed polymorphism shift is likely an integrated response to both thermal constraints and ecological demands, rather than solely a mechanism to regulate nest temperature. Consequently, *A. chartifex spiriti* appears to dynamically adjust its activity and foraging patterns, optimizing resource collection while minimizing the risks associated with environmental stress [73].

Thermoregulation, both active and passive, depends on the coordinated activities of individual workers to maintain the thermal homeostasis of the colony [10]. Our results indicate that *A. chartifex spiriti* employs a combination of complementary thermoregulatory mechanisms. Passively, the architecture of the nest, along with internal humidity gradients, heat dissipation capacity, and evaporative processes, plays a key role in buffering thermal fluctuations, particularly during periods of elevated ambient temperature.

Actively, worker behavior, expressed through alternations in foraging patterns along the nycthemeral cycle, contributes to internal heat input when external temperatures decrease. Thus, this study not only advances our understanding of thermal adaptations in arboreal ant species but also highlights the importance of the interplay between behavioral and architectural factors in the microclimatic regulation of nests. This integration represents a critical component of colony survival and ecological success under thermally challenging conditions. The findings presented here provide a robust foundation for future research on the ecological mechanisms that sustain biodiversity and functional stability in arboreal nesting systems.

## 5. Conclusions

This study deepens our understanding of thermoregulatory mechanisms of *A. chartifex spiriti*, highlighting the complementarity between nest architecture and worker behavior in maintaining the colony’s thermal homeostasis. We demonstrated that this species employs a combination of passive and active strategies to regulate internal nest temperature, ensuring the stability necessary for survival in tropical environments.

Further studies may explore how different arboreal ant species adjust their thermoregulatory mechanisms in response to more extreme environmental fluctuations, including: (i) prolonged droughts and heat waves; and (ii) seasonal changes and variations in rainfall regimes. Another promising direction involves examining the relationship between nest architectural variation and nesting strata to gain a better understanding of how these interactions contribute to colony resilience across ecosystems.

In summary, our findings emphasize the importance of investigating biodiversity and ecological processes in arboreal ant nesting systems, providing a strong foundation for future research aimed at the conservation and sustainable management of these complex ecological structures.

## Data Availability

The original contributions presented in this study are included in the article/Supplementary Materials. Further inquiries can be directed to the corresponding author.

## Supplementary Materials

The following supporting information can be downloaded at: https://www.mdpi.com/article/10.3390/insects17010032/s1, Table S1: Variation in the size of morphological traits of *Azteca chartifex spiriti* workers; Table S2: Pairwise multiple comparisons of morphological traits among different periods of the day in *Azteca chartifex spiriti* workers.
