# Supplementary material for: Thermal Ecology and Homeostasis in Colonies of the Neotropical Arboricolous Ant Azteca chartifex spiriti (Formicidae: Dolichoderinae)
Source: Insects. 2025 Dec 25;17(1):32. doi: 10.3390/insects17010032 (PMC12841854; doi:10.3390/insects17010032)
Supplement: Supplementary file 1 [file insects-17-00032-s001.zip › insects-4024264-supplementary.pdf]

Supplementary material

Table S1. Variation in the size of morphological traits of *Azteca chartifex spiriti* workers.

Period of day	Morphological Structure	Maximum Size (mm)	Minimum Size (mm)	Mean (±SD) (mm)
Morning	Head width	0.98	0.54	0.79 (0.12)
	Weber length	1.13	0.62	0.88 (0.12)
	Femur length	1.09	0.62	0.89 (0.11)
Afternoon	Head width	0.99	0.54	0.79 (0.12)
	Weber length	1.08	0.54	0.88 (0.12)
	Femur length	1.12	0.63	0.89 (0.12)
Night	Head width	1.0	0.51	0.87 (0.11)
	Weber length	1.14	0.61	0.96 (0.11)
	Femur length	1.10	0.62	0.95 (0.11)

Table S2. Pairwise multiple comparisons of morphological traits among different periods of the day in *Azteca chartifex spiriti* workers.

Morphological Structure	Period of day	*Z*	*P. unadj*	*P. adj*
Weber's length	Morning - Night	-5.0062963	<0.001	<0.001
	Morning - Afternoon	0.2509479	<0.001	<0.001
	Night - Afternoon	5.2708186	<0.001	<0.001
Head width	Morning - Night	-5.5548549	<0.001	<0.001
	Morning - Afternoon	-0.1105933	<0.001	<0.001
	Night - Afternoon	5.4382794	<0.001	<0.001
Femur length	Morning - Night	-4.5522361	<0.001	<0.001
	Morning - Afternoon	-0.1681527	<0.001	<0.001
	Night - Afternoon	4.3749876	<0.001	<0.001
